# Aggression towards shared enemies by heterospecific and conspecific cichlid fish neighbours

**DOI:** 10.1007/s00442-019-04483-0

**Published:** 2019-08-31

**Authors:** Topi K. Lehtonen

**Affiliations:** 1 Ecology and Genetics Research Unit, Faculty of Science, University of Oulu, Post Box 8000, 90014 Oulu, Finland; 2 School of Biological Sciences, Monash University, Monash, VIC 3800 Australia

**Keywords:** Aggression, Competition, Dear enemy, Heterospecific facilitation, Species coexistence

## Abstract

Successful territory defence is a prerequisite for reproduction across many taxa, and often highly sensitive to the actions of territorial neighbours. Nevertheless, to date, assessments of the significance of the behaviour of heterospecific neighbours have been infrequent and taxonomically restricted. In this field study, I examined the importance of both heterospecific and conspecific neighbours in a biparental fish, the convict cichlid, *Amatitlania siquia*. This was done by assessing the colonisation rates of vacant territories, the rates of aggression by the territory holders, and the overall rates of aggression towards intruders, in treatments that controlled the proximity of both neighbour types. Convict cichlid pairs colonised vacant nesting resources (territory locations) at similar rates independent of the proximity of heterospecific (moga, *Hypsophrys nicaraguensis*) or conspecific neighbours. However, a model of sympatric cichlid intruder was subjected to considerably higher overall levels of aggression when mogas were nearby. In contrast, the proximity of conspecifics did not have a significant effect on the overall aggression towards the intruder. These results suggest that previously demonstrated higher survival of convict cichlid broods in close proximity of mogas may be driven by aggression towards shared enemies. No conclusive evidence was found regarding whether mogas also influence convict cichlids’ investment into anti-intruder aggression: the results show a marginally non-significant trend, and a moderately large effect size, to the direction of a lower investment in mogas’, but not conspecifics’, proximity. More generally, heterospecific neighbours may provide protective benefits in a wider range of ecological settings than commonly considered.

**Electronic supplementary material:**

The online version of this article (10.1007/s00442-019-04483-0) contains supplementary material, which is available to authorized users.

## Introduction

Success in aggressive defence of a breeding territory towards rivals and would-be predators of offspring is often a prerequisite for reproduction. Territorial aggression, however, is costly due to the potential for injury, increased risk of predation, energy expenditure and/or time lost from foraging and other activities (Marler and Moore 1989; Jakobsson et al. 1995; Neat et al. 1998; Briffa and Elwood 2004). The costs of territoriality can reduce the population density at equilibrium and result in increased distances among individuals (López-Sepulcre and Kokko 2005). While the costs are likely to be affected by the proximity and identity of territorial neighbours, the presence of conspecifics can also entail benefits, a concept often referred to as the Allee effect (Courchamp et al. 1999; Stephens and Sutherland 1999). Beneficial neighbour effects may originate, for example, from anti-intruder aggression or predator satiation (Stephens and Sutherland 1999). For instance, in the cooperatively breeding cichlid fish, *Neolamprologus pulcher*, both large group size and high colony density significantly increase group persistence, with group size and density having interactive effects on reproductive output (Jungwirth and Taborsky 2015). Shelters suitable for breeding are occupied at a higher rate inside breeding colonies than at the colony edge, despite the availability of suitable habitat at the edge (Heg et al. 2008). Interestingly, any benefits of living in such dense groups may be linked not only to density-dependent decrease of predation risk but also reduced investment into anti-intruder behaviours (Daly et al. 2012; Jungwirth et al. 2015).

Similarly, intruder identity may affect the level of territorial aggression (Temeles 1994; Tibbetts and Dale 2007). For example, in the banded mongoose, *Mungos mungo*, residents respond less aggressively to scent marks of strangers than those of neighbouring packs (Müller and Manser 2007). A reduced level of aggression towards neighbours, so called ‘dear enemy’ effect, in turn, is widespread among animal taxa, such as mammals (Rosell et al. 2008; Zenuto 2010), birds (Hardouin et al. 2006; Briefer et al. 2008), reptiles (Fox and Baird 1992; Whiting 1999), amphibians (Jaeger 1981; Lesbarrères and Lodé 2002), fish (Aires et al. 2015; Sogawa et al. 2016) and insects (Pfennig and Reeve 1989; Dimarco et al. 2010). Indeed, an established neighbourhood may be associated with low costs, especially because of reduced aggression among well-established neighbours (Getty 1987; Temeles 1994). Rock pipits, *Anthus petrosus*, and male fiddler crabs of the genus *Uca* may even cooperate with their neighbours in territorial defence (Elfström 1997; Backwell and Jennions 2004; Detto et al. 2010).

To date, most studies investigating the significance of territorial relationships have focused solely on conspecifics (Temeles 1994; Tibbetts and Dale 2007), while heterospecific neighbour interactions have not been widely considered, although they can be similarly important (e.g. Forsman et al. 2002). Indeed, species differences in ecological, behavioural and morphological characteristics can enhance neighbour relationships in terms of decreased resource use overlap, transfer of useful information (Seppänen et al. 2007), higher foraging efficiency (Bshary et al. 2006), or wider-ranging predator detection and avoidance (Burger 1984; Semeniuk and Dill 2006). With regard to benefits arising from predation repellence by neighbours, individuals that aggressively defend their own territory may incidentally provide protection to territories of heterospecifics nearby (Krams et al. 2009; Campobello et al. 2012). For example, gulls (Wheelright et al. 1997; Väänänen 2000) and terns (Young and Titman 1986; Nguyen et al. 2006) are thought to offer protection from avian nest predators to other bird species nesting nearby. In addition, the stronger and more vigorous the individual(s) providing such protection, the higher the benefits to the nests in close proximity are likely to be: a predator can be expected to leave an area sooner, and have a lower success rate, when it is harassed intensively. The importance of the protector vigour is indirectly suggested, for instance, by predation rates on unguarded, ‘dummy’ nests being negatively correlated with aggressiveness of the female Eurasian hobby, *Falco subbuteo*, defending her own nest nearby (Bogliani et al. 1999).

To date, however, there has been very little quantitative evidence regarding intruders being actively chased away by the putative protective heterospecifics (Quinn and Ueta 2008). Furthermore, protection resulting from heterospecifics’ behaviour has been studied almost exclusively among birds or between birds and hymenoptera (Quinn and Ueta 2008). It is nevertheless possible that protective heterospecific interactions are also important in a range of other taxa. Benthic Lake Tanganyika cichlid fish, *Xenotilapia boulengeri*, can benefit from less frequent harassment by scale-eating cichlids, *Perissodus microlepis* and *Plecodus straeleni*, when staying in the proximity of aggressive substrate-brooding cichlids of the genus *Lepidiolamprologus* (Ochi and Yanagisawa 1998). Two species of closely related social cichlids with helper individuals, *Neolamprolgus pulcher* and *N*. *savoryi*, in turn, form colonies consisting of individuals of both species (Heg et al. 2008).

**Fig. 1:**
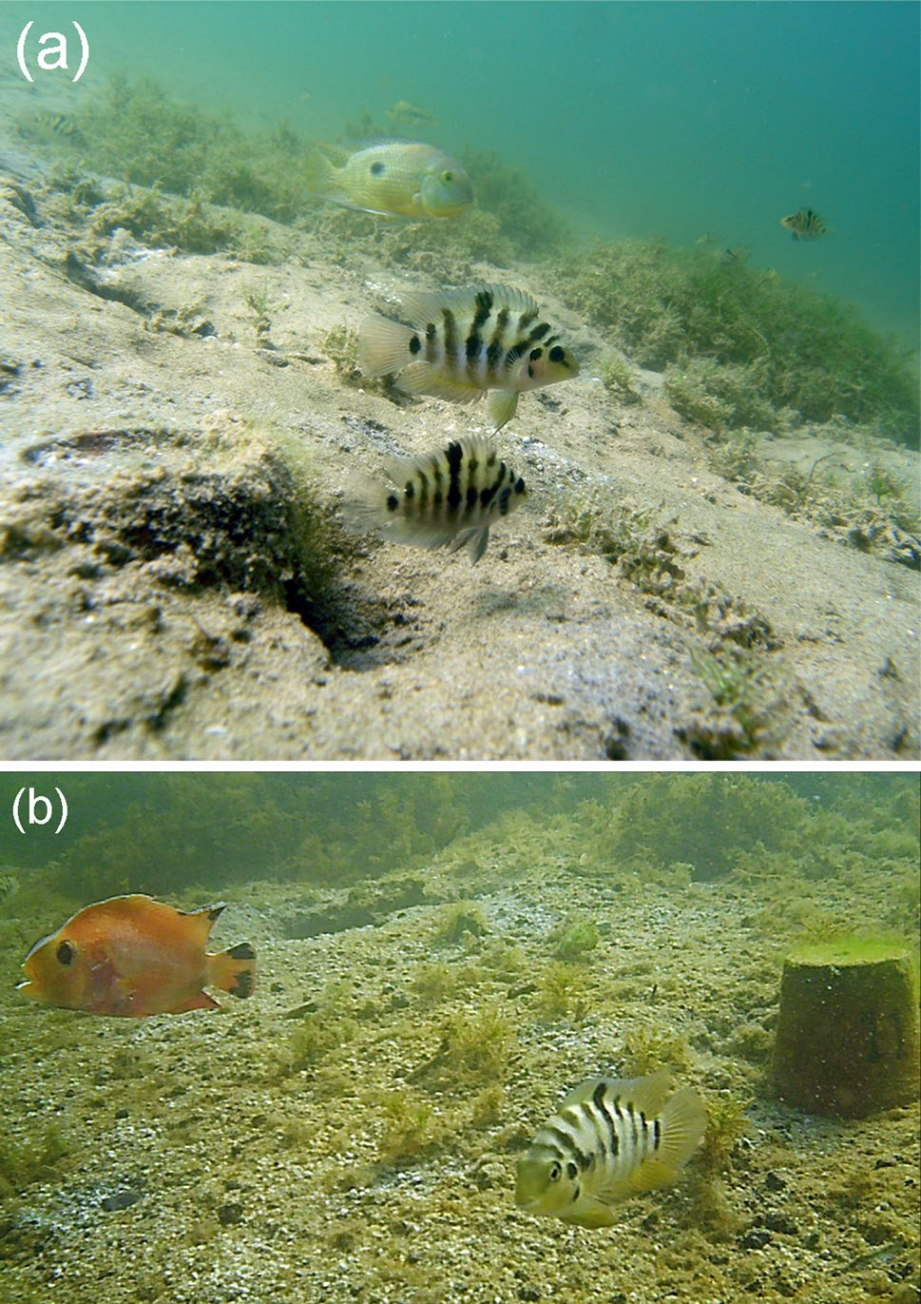
Convict cichlids occupying an artificial nesting resource and on the background **a** a moga male and non-breeding convict cichlids and **b** the intruder model

To investigate protective territorial aggression, and potential for energy savings with regard to heterospecific and conspecific neighbours, I focused on two species of Neotropical, territorial cichlid fish, convict cichlids, *Amatitlania siquia*, and mogas, *Hypsophrys nicaraguensis* (Fig. 1a). In both species, the male and female of a breeding pair claim a territory, which is then aggressively defended as a site for egg laying and later rearing the fry (McKaye 1977a; Lehtonen 2008; Lehtonen et al. 2015). Aggressive territory defence plays an essential role in the parental success in both species. In particular, competition for breeding territory sites, with both conspecific and heterospecific rivals, can be intense (McKaye 1977a; Lehtonen and Lindström 2008; Lehtonen et al. 2015), and predation on offspring and territorial takeovers are thought to be the primary causes of brood failure (McKaye 1977a, 1986; Lehtonen 2008). Interestingly, an earlier study showed that close proximity to a moga territory boosts the survival of convict cichlid broods (Lehtonen 2008). To date, however, mechanisms mediating the positive effects of mogas’ presence have not been assessed.

Here, I tested the hypothesis that aggressive territory defence by mogas could provide protection to territories of convict cichlids and especially to their broods. I also investigated the Allee effect, i.e. whether the proximity of conspecifics has comparable effects. I focused on the following three aspects of convict cichlids’ breeding territory acquisition and maintenance with respect to both moga and conspecific neighbours. First, to experimentally assess the rate of territory colonisation by convict cichlids in the presence of mogas and conspecifics, I placed vacant nesting resources (i.e. suitable territory locations), both close to, and farther away from, occupied heterospecific (moga) and conspecific (convict cichlid) territories. Second, I investigated whether more aggression is directed towards intruders close to the focal convict cichlid broods (territories) under the different neighbourhood scenarios (mogas and conspecifics either nearby or farther away). Finally, I tested the hypothesis that the focal convict cichlids might invest less energy into territory and brood defence when their territory is located close to a moga or conspecific territory.

## Materials and methods

The study consisted of three experiments, as detailed below, and it was conducted between November and December 2016, by scuba diving in Crater Lake Xiloá. The lake is located in western Nicaragua (latitude 12°12.8′N; longitude 86°19.0′W) and has moderately clear water (horizontal visibility during this study approximately 2–7 m). Convict cichlids occur in high numbers throughout the littoral zone of the lake, while apparently being limited by suitable nesting cavities that are required for successful reproduction and provide shelter for their eggs and fry (Lehtonen 2008; Lehtonen and Lindström 2008). Indeed, convict cichlid pairs readily accept an artificial nesting resource as the central structure within their territory and therefore the resources can be used to manipulate the locations of convict cichlid territories (Lehtonen 2008; Lehtonen and Lindström 2008; Fig. 1). Mogas are also common in the lake (Lehtonen et al. 2015) and typically excavate burrows in the substratum for the purpose of hiding their offspring (Lehtonen 2008; personal observations). Unlike those of convict cichlids, moga territories are not limited by pre-existing cavities that can be used for shelter. However, due to their larger body size (typical male standard length in mogas and convict cichlids: ~ 10 cm and ~ 5 cm, respectively; McKaye 1977a), reproduction of mogas may also be limited by availability of suitable (e.g. with regard to substratum type) territory space, which is often under competition with other cichlid species (McKaye 1977a, b; Lehtonen et al. 2015). Despite mogas being larger than convict cichlids, the two species use an overlapping niche space, and their juveniles, in particular, are likely to have very similar diets and shared would-be predators (McKaye 1977a; Lehtonen 2008; personal observations).

### Experiment 1

The aim of experiment 1 was to investigate whether vacant shelters (i.e. potential territory locations) are colonised faster by convict cichlid pairs when shelters are in close proximity to moga territories, as compared to those farther away from mogas. The experiment was initiated by manipulating the location of convict cichlid territories by placing a shelter either ‘close’ to (~ 50 cm), or ‘far’ from (150–180 cm), a moga territory (see Lehtonen 2008), at the depth of 2–3 m. Here, as well as in the two following experiments, I avoided placing shelters close to (within ~ 130 cm) any other fish territories. I randomised (using 1/100 s display of a waterproof stopwatch) whether a particular territory was assigned to the ‘close’ or ‘far’ treatment. As shelters, hereon called ‘nesting resources’, that are suitable for establishment of convict cichlid territories, I used clay flowerpots (maximum diameter: 8 cm, height: 6.5 cm) that had an entrance hole (~ 4 cm × 2.5 cm) on one side, and were turned upside down to rest on the substratum (see Lehtonen 2008). In contrast, moga territories in the study area were defined by excavations that the fish had dug in the sediment (Lehtonen 2008). The ‘close’ distance (~ 50 cm) was chosen to correspond to a typical radius of a moga territory (Lehtonen 2008; Lehtonen et al. 2015; Lehtonen and Wong 2017), as well as the low end of between-territory distances commonly observed in the wild (personal observations; see also Lehtonen et al. 2018).

After initiation of the experiment, nesting resources were checked once a day, 6–7 days a week, until they were colonised by convict cichlids. A nesting resource was considered to be colonised when a convict cichlid pair was defending eggs laid on the nesting resource’s inner surface (Lehtonen 2008; Lehtonen and Lindström 2008). In two cases, the nesting resource went missing after a few days (and before being colonised), and these replicates were, therefore, included in the survival analysis (see below) as ‘right censored’ data points (i.e. above a certain value but it remained unknown by how much; Lagakos 1979). One of the nesting resources was colonised by a species other than convict cichlids (poor man’s tropheus, *Hypsophrys nematopus*), and resulted in a disregarded replicate. Colonisation of nesting resources was assessed for *N* = 25 replicates (15 in ‘near’ and 10 in ‘far’ category).

Statistical analyses were conducted using R 3.3.2 software (R Development Core Team). To compare the colonisation rates between nesting resources close to, and far from, moga territories, I used Cox proportional hazard estimation (‘survival’ package in R). Each convict cichlid nesting location was used only once.

### Experiment 2

Experiment 2 complemented experiment 1 by adding the proximity of conspecifics as another factor that might explain the colonisation rate of vacant nesting resources (territory locations). Each replicate was initiated after the focal nesting resource in experiment 1 was colonised. At that point, a second nesting resource (identical to that of experiment 1, and hereon called the ‘secondary nesting resource’) was placed either close to (~ 50 cm), or far from (150–180 cm), the primary nesting resource of experiment 1. I again randomised (as described above) to which treatment each replicate was assigned. Importantly, the distance between the secondary nesting resource and the nearest moga territory was also kept at either ~ 50 cm (close) or 150–180 cm (far), resulting in a 2 × 2 design with regard to the proximity and species of the neighbours. As above, when placing the nesting resources, close proximity of cichlid territories, other than those of the focal (i.e. nearest) moga and conspecific pairs, was avoided. In some cases, the focal moga territory either failed or the moga parents relocated their offspring during the replicate (see Lehtonen 2008). In such cases, the replicate was considered to be in the ‘far from moga’ category, as long as another moga territory was located within 130–260 cm from the secondary nesting resource (*N* = 3).

If the replicate needed to be terminated for logistic reasons (time constraints, a break in air tank availability) before the secondary nesting resource was colonised, the replicate was included as a right censored data point in the analysis (Lagakos 1979). The number of days until the secondary nesting resource was colonised, in relation to the proximity of the nearest moga territory (close versus far) and nearest conspecific territory (close versus far), as well as their interaction, was assessed using Cox proportional hazard estimation (‘survival’ package). Colonisation of *N* = 21 nesting resources was assessed.

### Experiment 3

In experiment 3, I assessed the rate of aggression directed to an intruder model, which was placed next to the focal convict cichlid territory that was associated with a nesting resource (see above) and occupied by a convict cichlid pair. In particular, to have control over intruder approaches, I presented the focal convict pair with a ‘dummy’ model of a sympatric cichlid fish, *Amphilophus sagittae* (as per Lehtonen 2017; Lehtonen and Wong 2017; Fig. 1b). This intruder species was chosen because it shares the same breeding habitat with both mogas and convict cichlids, and it is their potential territory space competitor and/or predator of their offspring (McKaye 1977a; Lehtonen et al. 2015). *Amphilophus sagittae* has two colour morphs, of which I used the ‘gold’ morph, because an earlier study (Anderson et al. 2016), and pilot trials conducted in early November 2016, suggested that convict cichlids are markedly more responsive towards brighter coloured opponents. Similarly, mogas have been found to be up to 50% more aggressive towards the gold than the dark colour morph (Lehtonen et al. 2015). More generally, hand-made intruder models have been very useful for measuring rates of aggression in cichlids (Cravchik and Pazo 1990; Barlow and Siri 1994; Ochi and Awata 2009), including the convict cichlid (Beeching et al. 1998; Anderson et al. 2016) and moga (Lehtonen et al. 2015; Lehtonen and Wong 2017). The intruder model used in the current study was made by glueing a waterproof, photographic colour print of the lateral side of an adult *A*. *sagittae* (sex unknown) onto each lateral side of an elliptical floating plate (thickness: 6 mm). The model was 16 cm long and attached to a small sinker with a thin, transparent fishing line (following Lehtonen 2017; Lehtonen and Wong 2017), so that it floated in a natural position approximately 10 cm above the substratum (Fig. 1b).

**Fig. 2:**
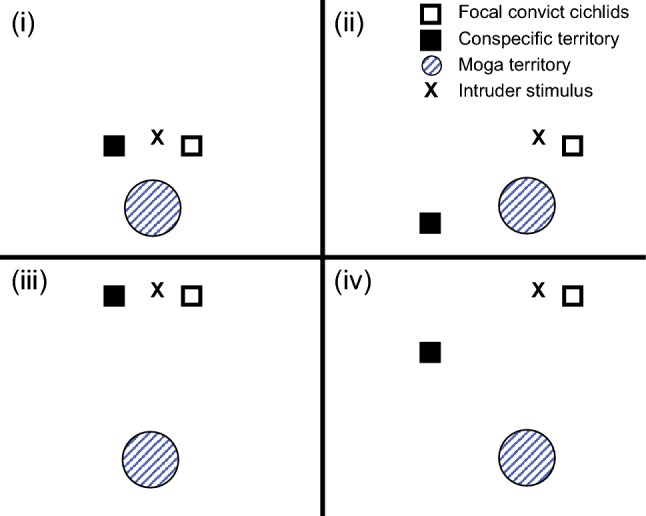
Schematic top view of the different treatments in experiment 3 (*N* = 22). The different treatments were: (i) the nearest moga and conspecific territories close (*N* = 3); (ii) moga close, conspecific far (*N* = 6); (iii) moga far, conspecific close (*N* = 7); (iv) both moga and conspecific territories far (*N* = 6)

To start an aggression assessment trial, I placed the intruder model at a distance of ~ 35 cm from the focal convict cichlid territory (Fig. 2). Despite the focal territory being associated with a nesting resource, for practical reasons, the distance between the intruder model and territory was defined by the location of the brood (as per Lehtonen 2017). The focal territory, in turn, was located either close (~ 50 cm) or far (150–180 cm) in relation to the nearest conspecific territory (also associated with a nesting resource), as well as the nearest moga territory, resulting in a 2 × 2 design. The treatment to which each replicate was assigned was randomised, as described above. The model intruder was placed so that it was clearly visible from the directions of all of three territories (focal convict cichlid, treatment convict cichlid and treatment moga), while closest to the focal territory (Fig. 2). I then counted the number of aggressive responses by the focal pair, any mogas, and all other, miscellaneous fish (together allowing assessment of the overall rate of aggression), towards the intruder model for 5 min. Such counts of aggressive responses have, in some previous studies, been referred to as the ‘total aggression rate’ (sensu Lehtonen et al. 2012; Oldfield et al. 2015). Typical aggressive responses involved either a rapid advance (often followed by a bite and a retreat), or slow movement towards the intruder model with pronouncedly flared gill covers and fins. Experiment 3 (*N* = 22 replicates) was conducted 1–9 days (mean ± S 6.6 ± 0.5) after colonisation of the focal nest.

The response variables (i.e. counts of aggression) were checked for normality (with shapiro.test function; Royston 1995) and homogeneity of variance (with bartlett.test function; Bartlett 1937), as well as visually by plotting the residuals. After square root transformation, it was appropriate to apply general linear models (‘lm’ function in R) for the analyses of the overall aggression rate displayed by all fish and aggression rate displayed by the focal convict cichlids. Aggression rate by all mogas, in turn, was analysed using a negative binomial distribution, as appropriate for overdispersed count data (‘glm.nb’ function in R). In all standalone models [i.e. (i) overall aggression by all fish, (ii) aggression by focal convict cichlids—used as a proxy of their workload and (iii) aggression by mogas], the distance to the closest mogas (close/far) and convict cichlid neighbours (close/far), as well as the interaction between these two factors, were denoted as explanatory variables. To minimise the effect of the number of days after the focal nest was colonised, it was added as a covariate. If the interaction between the proximity of neighbouring mogas and conspecifics was found to be non-significant, I then refitted the model without the interaction, before interpreting the main effects (as per Crawley 2007).

## Results

### Experiment 1

There was no significant difference in the colonisation rates of nesting resources placed close to, versus farther away from, moga territories (Cox proportional hazard estimation, *z* = 1.091, *P* = 0.28).

### Experiment 2

The moga × conspecific neighbour proximity interaction did not have a significant effect on the colonisation rate of the secondary nesting resource (Cox proportional hazard estimation, *z* = 0.401, *P* = 0.69). Refitted without the interaction effect, the model indicated that the colonisation rate of the secondary nest was not significantly affected by the distance to the nearest moga territory (Cox proportional hazard estimation, *z* = 1.096, *P* = 0.27). Similarly, the proximity of the nearest conspecific territory did not have a significant effect on the colonisation rate (Cox proportional hazard estimation, *z* = 0.374, *P* = 0.71).

### Experiment 3

1. Overall aggression by all fish: after removal of the non-significant interaction term (*F*_1,17_ = 0.9823, *P* = 0.34), the simplified model showed that the overall rate of aggression (by all fish) towards an intruder model was significantly higher when there was a moga territory in close proximity than when the closest moga territory was farther away (linear model, *F*_1,18_ = 17.09, *P* < 0.001; Fig. 3a), whereas proximity of a conspecific territory (linear model, *F*_1,18_ = 0.7522, *P* = 0.40; Fig. 3b) and the covariate (linear model, *F*_1,18_ = 2.671, *P* = 0.12) did not have a significant effect.

**Fig. 3:**
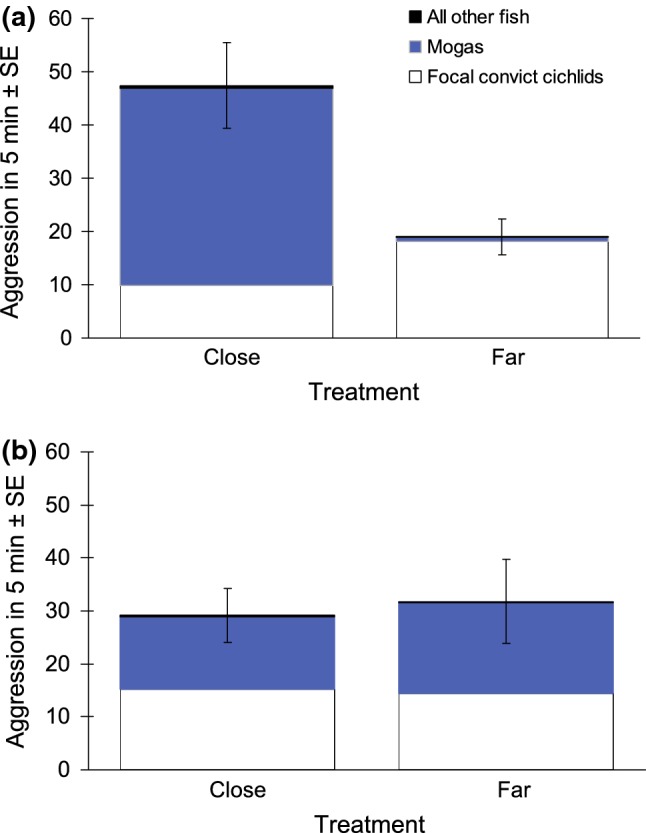
The overall numbers of aggressive responses towards a model intruder placed next to a convict cichlid territory, with a breakdown by the aggressor group. The intruder model, as well as the focal convict cichlid territory, was either in close proximity to, or farther away from, **a** the nearest moga territory (*N*_Close_ = 9, *N*_Far_ = 13) and **b** the nearest conspecific territory (*N*_Close_ = 10, *N*_Far_ = 12)
2. Aggressive responses by the focal convict cichlid pair: the moga × conspecific neighbour interaction term had a marginally non-significant effect (*F*_1,17_ = 4.043, *P* = 0.060). When the interaction was removed before interpreting the main effects (as per Crawley 2007), the proximity of a moga territory had a marginally non-significant effect: the focal convict cichlid pair tended to display a lower rate of aggression in mogas’ presence (*F*_1,18_ = 3.552, *P* = 0.076; Fig. 3a). Neither the proximity of a conspecific territory (*F*_1,18_ = 0.0565, *P* = 0.81; Fig. 3b) nor the covariate (*F*_1,18_ = 0.7522, *P* = 0.40) had a significant effect. The conclusions remained the same if the marginally non-significant interaction term was retained in the model: moga effect (*F*_1,17_ = 4.153, *P* = 0.057), conspecific effect (*F*_1,17_ = 0.0661, *P* = 0.80), the covariate (*F*_1,17_ = 0.8829, *P* = 0.36).
3. Aggression by mogas: I first removed the non-significant interaction term (general linear model, *z* = 0.00, *P* = 1.0). The simplified model showed that the proximity of moga neighbours had a highly significant effect (*z* = 9.267, *P* < 0.001): the rate of aggression by mogas directed to the intruder was much higher when there was a moga territory in the vicinity of the focal convict cichlid territory (Fig. 3a). This aggression was displayed almost exclusively by the nearest moga territory holders. The proximity of the neighbouring convict cichlid territory (i.e. focal convict cichlids’ conspecifics) also had an effect (*z* = 2.057, *P* = 0.040), with mogas displaying a lower rate of aggression towards the intruder when a neighbouring convict cichlid territory was close (Fig. 3b). The covariate did not have a significant effect (*z* = 0.832, *P* = 0.41).

## Discussion

An intruder model placed next to a convict cichlid territory was subjected to more than twice the number of aggressive responses per unit time when there was also a moga territory in close proximity than when the nearest moga territory was farther away. Manipulation of the proximity of the nearest conspecific (i.e. convict cichlid) territory, in turn, did not significantly affect the overall rate of aggression directed to the intruder. Interestingly, mogas’ share of the overall rate of aggression towards the intruder was considerable and much higher when the moga territory was close to the focal convict cichlid territory than when farther away from it. These results support the hypothesis that aggression by mogas towards shared territorial intruders benefits convict cichlids. In particular, convict cichlids that have a territory close to that of mogas not only have a higher brood survival (Lehtonen 2008), but an intruder of a convict cichlid territory is also subjected to higher overall levels of aggression in mogas’ proximity. Most of that higher level of overall aggression is displayed by the neighbouring mogas. More generally, the results provide evidence for protective heterospecific associations among taxa other than certain bird species (see Quinn and Ueta 2008). Most previous studies have provided only anecdotal evidence for attacks towards predators by the more aggressive species (‘protector’), and intensities of territory defence have rarely been assessed (Quinn and Ueta 2008). The current results are, therefore, significant in demonstrating the intensity of such aggression-mediated protective associations in a heterospecific context, and how this can take place in non-avian species, such as fish. This is relevant especially because predation is a strong selective pressure across a range of different taxa and commonly one of the leading causes of mortality in prey species (Almany and Webster 2006; Lima 2009).

The result that the proximity of conspecifics did not have a significant effect on the overall rate of aggression directed to an intruder implies that conspecific convict cichlids provide much less protection against brood predators than mogas. In addition, the costs of close neighbour proximity might be higher in the case of conspecifics than mogas, due to intraspecific aggression and sexual competition. Indeed, an earlier study suggests that nesting resources that are sparsely distributed are more popular among convict cichlids than more aggregated nesting resources (Lehtonen and Lindström 2008). The phase of convict cichlids’ territory establishment, with regard to the number of days from territory colonisation, did also not have a significant effect on any of the aggression rates.

Energy savings in relation to anti-predator behaviours have recently been suggested to be among the main benefits of living in conspecific groups (Daly et al. 2012; Jungwirth et al. 2015). In addition, territory owners of some species adjust the level of their territorial aggression according to the perceived local predation pressure (Graw and Manser 2007; Kaplan et al. 2009; Dutour et al. 2016). Here, focal convict cichlid pairs exhibited 84% higher level of territorial aggression towards an intruder when their territory was ‘farther away from’, as compared to ‘closer to’ the nearest moga territory (Fig. 3). In the case of the proximity of the nearest conspecific territory, the effect was only 6% and in the opposite direction (Fig. 3). Whether the proximity of mogas does indeed result in any energy savings remained unresolved: the moderately high difference in the level of convict cichlids’ aggression close to, versus farther away from, mogas was marginally non-significant. In addition, if intruder pressure is particularly high close to moga territories, any energy savings to convict cichlids nearby, due to mogas’ aggressiveness, could be negated. Such a scenario is unlikely, however, because there were no noticeable differences in the numbers of potential intruders approaching moga territories versus adjacent territories or areas (personal observations). Convict cichlid broods also have a higher survival when close to moga territories (Lehtonen 2008), further suggesting that intruder pressure is not more intense close to mogas.

Competitive interactions among conspecifics might affect alertness or prioritisation of aggression by focal convict cichlids (see Lehtonen and Lindström 2008). Similarly, if convict cichlids perceive territories close to mogas as of particularly high value, they might be expected to defend their broods at such sites more aggressively (Arnott and Elwood 2008). However, proximity of conspecifics did not have a significant effect on the rate of aggression by focal convict cichlids, and if anything, convict cichlids displayed less aggression to an intruder when a moga territory was close by, presumably due to mogas’ protective aggression. In a similar fashion, mogas directed less aggression to an intruder when there was another convict cichlid territory close to the focal one.

Convict cichlid pairs did not colonise nesting resources at different rates with respect to proximity of mogas or conspecifics. The lack of a positive moga effect might be seen surprising, given the benefits associated with mogas’ presence (Lehtonen 2008; the current study). Similarly, one might have expected a negative effect by the presence of conspecifics, because of the high level of their intraspecific aggression, and an earlier study showing that nesting resources are more popular among convict cichlids when sparsely spaced rather than aggregated (Lehtonen and Lindström 2008). What might have been the reasons for the lack of any treatment effects in the rate of colonisation of nesting resources? First, territory-holding mogas, as well as conspecifics, are very aggressive towards convict cichlids that are not their established neighbours (Lehtonen 2008; personal observations), which may make the establishment of a territory in close proximity to moga (and conspecific) neighbours difficult. In this respect, the presence of mogas is likely to result not only in benefits but also costs, due to their aggressiveness and/or competition for resources (as suggested by McKaye 1977a). Second, because nest colonisation was based on the presence of eggs (as per Lehtonen 2008; Lehtonen and Lindström 2008), the colonisation rate may have been similarly limited across all treatments, by either pair formation or convict cichlid females’ readiness to lay eggs. Third, if quality of territories varies for reasons other than the presence of mogas or conspecifics (for instance, because of non-standard distances to the closest territories of other fish), detection of any treatment differences could be more difficult.

Thus far, the association between convict cichlids and mogas has only been examined from the point of view of the former. However, it is likely that convict cichlid breeding pairs are not particularly costly neighbours to mogas, as suggested by the smaller natural minimum distances between moga and convict cichlid territories than adjacent moga territories (personal observations), and the relatively low rates of aggression between established moga and convict cichlid neighbours (Lehtonen 2008). More broadly, the costs and benefits of having a territory in close proximity to a neighbour may be context dependent. For instance, great tits, *Parus major*, prefer to nest in trees with ants—which may pose a danger of injury for both predators as well as the nesting great tits—in areas of high, but not low, predator abundance (Haemig 1999). Such context dependency of heterospecific associations may have contributed to the research focus, to date, having been biased towards conspecifics and a limited range of taxa. In this respect, the findings of the current study suggest that valuable avenues for future research include consideration of a diverse array of animal groups, precise quantifications of (protective) heterospecific interactions and comparisons of the interactions in the presence versus absence of both heterospecific and conspecific neighbours. Indeed, by influencing reproductive success in key species, protective heterospecific interactions may have important community level implications (see Bruno et al. 2003).

To conclude, this study shows that a model intruder placed close to a convict cichlid territory was subjected to considerably higher levels of aggression when there was a moga territory in close proximity than when the nearest moga territory was farther away. The proximity of a conspecific territory did not have an effect. With regard to potential for energy savings in anti-intruder aggression, only weak support was found in close proximity to mogas and none near to conspecifics. Moreover, vacant nesting resources were colonised at similar rates, independent of the proximity of the nearest moga or conspecific territory. Overall, these results are consistent with the previous finding of a higher brood success in the proximity of moga territories (Lehtonen 2008), by suggesting that the improved brood survival could result from aggression towards shared intruders by territorial mogas. Hence, the results underscore the importance of considering and quantifying protective interactions in a diverse array of taxa.

## Electronic supplementary material

Below is the link to the electronic supplementary material.

Supplementary material 1 (DOC 152 kb)
