# Supplementary material for: Aggression towards shared enemies by heterospecific and conspecific cichlid fish neighbours
Source: Oecologia. 2019 Aug 31;191(2):359–68. doi: 10.1007/s00442-019-04483-0 (PMC6763407; doi:10.1007/s00442-019-04483-0)
Supplement: Supplementary file 1 — Supplementary material 1 (DOC 152 kb) [file 442_2019_4483_MOESM1_ESM.doc]

***Supplementary Material***

**Aggression toward shared enemies by heterospecific and conspecific cichlid fish neighbours**

Topi K. Lehtonen^1,2^

^1^Ecology and Genetics Research Unit, Faculty of Science, University of Oulu, Post Box 8000, 90014 Oulu, Finland

^2^School of Biological Sciences, Monash University, VIC 3800, Australia

**Data experiment 1**

| Nest ID | Treatment | Days | Censor | Depth |
| --- | --- | --- | --- | --- |
|  |  | passed |  |  |
| P19 | Far | 3 | 1 | 2.3 |
| P55 | Near | 8 | 1 | 2 |
| P71 | Near | 2 | 1 | 2.4 |
| P35 | Near | 2 | 1 | 2.1 |
| N47 | Near | 5 | 1 | 2.3 |
| P1 | Near | 4 | 1 | 2.5 |
| P54 | Far | 2 | 1 | 2.8 |
| P76 | Near | 4 | 1 | 2.3 |
| N10 | Far | 3 | 1 | 2.6 |
| P2 | Near | 16 | 1 | 2.3 |
| P17 | Far | 2 | 1 | 2.4 |
| P22 | Far | 2 | 0 | 2.4 |
| N05 | Near | 2 | 1 | 2.4 |
| P20 | Near | 11 | 1 | 2.3 |
| P3 | Far | 2 | 1 | 2.4 |
| P29 | Near | 2 | 1 | 2.5 |
| P61 | Near | 5 | 1 | 2.3 |
| P16 | Far | 5 | 1 | 2.8 |
| P46 | Near | 9 | 1 | 2.6 |
| P15 | Near | 3 | 1 | 3 |
| P14 | Far | 6 | 1 | 2.8 |
| P12 | Far | 4 | 0 | 2.5 |
| P12 | Near | 5 | 1 | 2.5 |
| N11 | Near | 3 | 1 | 2.4 |
| N42 | Far | 2 | 1 | 2.3 |

**Data experiment 2**

| Nest ID | Moga | Convict | Days | Censor | Depth |
| --- | --- | --- | --- | --- | --- |
|  | treatment | treatment | passed |  |  |
| N13B | Near | Near | 5 | 1 | 2.2 |
| N25B | Near | Near | 6 | 1 | 2.5 |
| P56B | Near | Far | 5 | 1 | 2 |
| P15B | Near | Near | 3 | 1 | 2.3 |
| P37B | Far | Near | 5 | 1 | 2.5 |
| G31B | Far | Near | 7 | 1 | 2.7 |
| P27B | Near | Far | 2 | 0 | 2.1 |
| P44B | Far | Far | 3 | 0 | 2.6 |
| P7B | Near | Far | 1 | 1 | 2.3 |
| P41B | Far | Far | 8 | 1 | 2.5 |
| N82B | Near | Near | 4 | 1 | 2.4 |
| P32B | Near | Far | 5 | 1 | 2.3 |
| P23B | Far | Near | 7 | 1 | 2.5 |
| N62B | Near | Far | 8 | 0 | 2.5 |
| P16B | Near | Far | 2 | 1 | 2.4 |
| P45B | Far | Far | 4 | 1 | 2.8 |
| P48Ba | Near | Far | 2 | 1 | 2.9 |
| N24B | Far | Far | 3 | 1 | 2.9 |
| P46B | Near | Near | 3 | 0 | 2.4 |
| N93B | Near | Near | 2 | 1 | 2.4 |
| P48Bb | Far | Far | 4 | 0 | 2.6 |

**Data experiment 3**

| Nest ID | Moga | Convict | Focal convict | Nearest | All | Other | Overall | Days since | Depth |
| --- | --- | --- | --- | --- | --- | --- | --- | --- | --- |
|  | treatment | treatment | cichlid | moga | moga | fish | aggression | colonisation |  |
| N10P1 | Far | Far | 8 | 0 | 0 | 0 | 8 | 9 | 2.6 |
| P44P2 | Far | Far | 24 | 0 | 0 | 0 | 24 | 2 | 2.6 |
| P3P1 | Far | Near | 31 | 0 | 0 | 0 | 31 | 9 | 2.4 |
| P15P1 | Near | Far | 5 | 15 | 15 | 0 | 20 | 7 | 3 |
| P35P1 | Near | Far | 24 | 16 | 16 | 0 | 40 | 7 | 2.1 |
| P14P1 | Far | Far | 6 | 0 | 0 | 0 | 6 | 6 | 2.8 |
| P71P1 | Far | Near | 25 | 0 | 0 | 1 | 26 | 8 | 2.4 |
| P1P1 | Far | Near | 12 | 0 | 2 | 0 | 14 | 9 | 2.5 |
| P54P1 | Far | Near | 21 | 0 | 2 | 0 | 23 | 8 | 2.8 |
| P29P1 | Near | Far | 4 | 41 | 41 | 0 | 45 | 9 | 2.5 |
| P61P1 | Near | Far | 30 | 8 | 8 | 2 | 40 | 7 | 2.3 |
| P19P1 | Near | Near | 4 | 58 | 58 | 0 | 62 | 8 | 2.3 |
| N47P1 | Near | Near | 4 | 32 | 32 | 2 | 38 | 4 | 2.3 |
| P17P1 | Far | Far | 4 | 0 | 0 | 0 | 4 | 6 | 2.4 |
| P41P2 | Far | Far | 38 | 0 | 0 | 0 | 38 | 1 | 2.5 |
| N05P1 | Near | Near | 3 | 36 | 37 | 0 | 40 | 8 | 2.4 |
| P20P1 | Near | Far | 5 | 31 | 31 | 0 | 36 | 5 | 2.3 |
| P23P2 | Far | Near | 6 | 0 | 1 | 0 | 7 | 8 | 2.5 |
| P16P2 | Near | Far | 10 | 95 | 95 | 0 | 105 | 4 | 2.4 |
| N24P2 | Far | Far | 15 | 0 | 0 | 0 | 15 | 8 | 2.9 |
| N11P1 | Far | Near | 36 | 0 | 2 | 0 | 38 | 7 | 2.4 |
| G31P2 | Far | Near | 11 | 0 | 2 | 0 | 13 | 5 | 2.7 |
